# Characterization, Cytotoxicity and Anti-Inflammatory Effect Evaluation of Nanocapsules Containing Nicotine

**DOI:** 10.3390/bioengineering8110172

**Published:** 2021-11-03

**Authors:** Carolina Landau Albrecht, Laura Elena Sperling, Daikelly Iglesias Braghirolli, Patricia Pranke

**Affiliations:** 1 Hematology and Stem Cell Laboratory, Faculty of Pharmacy, Universidade Federal do Rio Grande do Sul (UFRGS), Porto Alegre 90.610-000, RS, Brazil; daikellyib@gmail.com (D.I.B.); patriciapranke@ufrgs.br (P.P.); 2 Postgraduate Program in Physiology, UFRGS, Porto Alegre 90.610-000, RS, Brazil; 3 Stem Cell Research Institute (Instituto de Pesquisa com Células-Tronco), Porto Alegre 90.610-000, RS, Brazil

**Keywords:** nanocapsules, Eudragit, nicotine, inflammation, PC12

## Abstract

(1) Background: Nanotechnology is an emerging field that can be applied in the biomedical area. In this study, Eudragit nanocapsules (NCs) containing nicotine were produced. Nicotine is the main alkaloid found in tobacco and has anti-inflammatory properties. NCs containing nicotine may be used as an adjuvant therapy in the treatment of inflammation in the central nervous system. (2) Methods: Nanocapsules were prepared by the interfacial deposition of the pre-formed polymer method and characterized in terms of zeta potential, diameter, polydispersity index, pH, encapsulation efficiency (EE), stability and sustained release profile. In vitro tests with the PC12 cell line were performed, such as MTT, LIVE/DEAD and ELISA assays, to verify their cytotoxic and anti-inflammatory effects. (3) Results: The nanocapsules presented satisfactory values of the characterization parameters; however, poor encapsulation was obtained for nicotine (8.17% ± 0.47). The in vitro tests showed that the treatment with nanocapsules reduced cell viability, which suggests that the Eudragit or the amount of polymer on top of the cells may be detrimental to them, as the cells were able to survive when treated with bulk nicotine. ELISA showed an increment in the expression of IL-6 and IL-1β, corroborating the hypothesis that NCs were toxic to the cells because of the increase in the levels of these pro-inflammatory cytokines. (4) Conclusions: This study demonstrates that NCs of Eudragit present toxicity. It is therefore necessary to improve NC formulation to obtain better values for the encapsulation efficiency and reduce toxicity of these nanodevices.

## 1. Introduction

Nanotechnology is an emerging interdisciplinary field that combines chemistry, physics, engineering and biology and has been extensively used in recent years in the health research area. It aims to create functional materials by controlling the matter in the nanometer scale, thus, producing materials with new chemical, physical and biological properties. It can be used particularly in the biomedical area to produce nanodevices or nanorobots, which are useful in diagnostic procedures; nanotechnology is also used to produce scaffolds or matrices utilized in regenerative medicine to mimic the extracellular matrix [1,2]. In addition, they can be employed to improve the biocompatibility of biological systems such as prosthetics, and finally, nanotechnology is extremely useful for the production of sustained release devices for pharmaceutical compounds [1].

Nanometrical devices of sustained release present several advantages compared to conventional administration forms, such as pills, injections, patches and sprays. Among these advantages, some of them include better therapeutic efficacy; reduction of side effects and thus, toxicity reduction; reduction of early inactivation of drugs; better targeting of the drug to the target tissue; ease of transposing biological barriers; and greater versatility, as it is possible to encapsulate lipophilic and hydrophilic compounds, among others [1]. Besides this, sustained release devices generally need only one dosage to reach the desired drug concentration. There are a variety of devices today, but the main ones are the so-called nanoparticles. Nanoparticles can be classified as nanospheres (matricial systems, with no nuclear compartment, where the drug is dissolved in the whole matrix), and nanocapsules (reservoir systems, containing a differentiated nucleus where the drug can be encapsulated) [3]. The nanocapsules are organized as a lipid core surrounded by a polymer wall.

Eudragit is the brand name for various polymethacrylate-based copolymers that includes anionic, cationic and neutral copolymers, based on methacrylic acid and methacrylic/acrylic esters or their derivatives [4]. These polymers are used as coating materials in pharmaceutical preparations for the purpose of achieving a better or controlled release [4]. Nanocapsules of Eudragit are produced and typically administered enterally and their applications include drug release for colon cancer [5], inflammatory bowel disease [6], release of antioxidant and anti-inflammatory substances [7].

Nanotechnology holds great promise in the development of new therapeutic strategies to diagnose, treat or even regenerate the nervous system in neurological disorders, such as multiple sclerosis, Parkinson’s disease, Alzheimer’s disease and spinal cord injury (SCI) [8]. Nanocapsules are excellent candidates for enhancing the drug bioavailability for the central nervous system [9].

The NF-kB pathway is crucial in the inflammatory response because of the induction of the expression of a series of pro-inflammatory genes, including those which encode chemokines and cytokines, such as the pro-inflammatory cytokines TNF-α, IFN-γ and IL-1β. Under physiologic conditions, nuclear transcription factor NF-kB induces the transcription of more than five hundred genes, regulating survival, activation and differentiation of cells of the innate immune system and inflammatory T cells, among other functions [10].

Nicotine is the main alkaloid found in tobacco and, in the human organism, it has the capacity of binding to acetylcholine nicotinic receptors, which leads to the release of a variety of neurotransmitters such as noradrenaline, dopamine, acetylcholine, serotonin, GABA, glutamate and endorphins [11]. Furthermore, nicotine has been extensively studied due to its anti-inflammatory potential. Although it has deleterious effects when associated with cigarette smoking, researchers have shown benefits of nicotine in modulating inflammatory diseases such as ulcerative colitis, Parkinson’s disease and Alzheimer’s disease [12]. It is of significance that previous studies have shown that it can suppress NF-kB transcription factor through binding to the nicotinic receptors, specifically the α7nAChR subtype. Moreover, it is suggested that the α7 subunit is crucial in the regulation of the inflammatory process [13]. It is believed that nicotine, after binding to the α7nAChR receptor, acts through a number of pathways that lead to NF-kB repression, and the down-regulation of pro-inflammatory cytokines expression [12].

Besides inhibiting the pro-inflammatory NF-kB pathway, various studies in the literature show the immunomodulatory effect of nicotine, suggesting that it can suppress T cell and B cell development and activation, and decrease T cell response, altering differentiation, phenotype and function of antigen presenting cells (APCs), such as macrophages and dendritic cells [14]. Lee et al. also demonstrated in a compressive spinal cord injury model that nicotine could attenuate the enzyme inducible Nitric Oxide Synthase (iNOS), an important pro-inflammatory agent [15]. Moreover, Kaur and colleagues showed that nicotine was neuroprotective against excitotoxicity, another important hallmark of SCI secondary trauma [16].

In the present work, it was hypothesized that nicotine could be an adjuvant in the treatment of CNS diseases by reducing neuroinflammation. Therefore, a new strategy composed of polymeric nanocapsules containing nicotine has been produced and characterized. Additionally, the cytotoxic and anti-inflammatory effects of the nanocapsules containing nicotine were evaluated in vitro using the cellular neural model PC12 cell line.

## 2. Materials and Methods

### 2.1. Preparation of Eudragit Nanocapsules Containing Nicotine

The nanocapsules were produced by the interfacial deposition of the pre-formed polymer method [3]. Initially, an emulsion was prepared by mixing an organic phase to an aqueous phase. The aqueous phase contained 26 mL of distilled water and 0.038 g of the detergent Triton X-100. The organic phase was prepared with 0.05 g of the cationic polymer (Eudragit^®^ RL, Evonik, São Paulo, SP, Brazil), 15 mg of nicotine ditartrate dihydrate (Acros Organics^®^, São Paulo, Brazil) 0.082 g of castor oil and 13.5 mL of acetone. Upon homogenization of the phases, the organic phase was poured into the aqueous phase, under vigorous magnetic stirring for 10 min. Following this, the excess solvent and water were evaporated in a rotary evaporator at 30 mbar and 40 °C bath heating in order to adjust the concentration of nicotine and reach the final volume of 5 mL of suspension.

Empty nanocapsule suspensions were prepared by the same method, without adding nicotine to the formula.

### 2.2. Nanocapsule Characterization

#### 2.2.1. Structural Analysis

The morphological characteristics of the prepared nanocapsules were evaluated by Transmission Electron Microscopy (TEM) (JEM 1200 Exll, Peabody, MA, USA) after preparation with negative contrast.

#### 2.2.2. Determination of pH

The pH was determined at room temperature, using pH strips.

#### 2.2.3. Nanocapsule Diameter and Zeta Potential

The average size of the nanocapsules and the respective polydispersity index (PdI) were determined by the dynamic light scattering method (ZetaSizer Nano ZS, Malvern Instruments, Worcestershire, UK). The samples were prepared by diluting the suspensions in sodium chloride (1 mM) at 1:100. Zeta potential was determined using the same equipment and the same samples.

#### 2.2.4. Determination of Nanocapsule Nicotine Content and Encapsulation Efficiency

The nicotine content of the nanocapsules was determined by the High-Performance Liquid Chromatography (HPLC) method. The sample was diluted in 1 mL of methanol and incubated at room temperature overnight (18 h). Following this, the samples were filtered and injected in the HPLC system (Dionex Ultimate 3000, São Paulo Brazil equipped with the Dionex Acclaim 120 C8 column) for analysis. The following parameters were used: wavelength 259 nm, temperature 30 °C, injected volume 20 μm, mobile faze A water, mobile faze B methanol, and a flux 0.5 mL/min. For determining the encapsulation efficiency, the samples were filtered in microfiltration tubes composed of 0.1 μm pore membranes (MilliPore) by centrifugation (Thermo Scientific, SL8R, São Paulo, Brazil) for 2 min at 6000 rpm, and the supernatants were collected. Nicotine content in the supernatants (free nicotine) was also determined by HPLC. The encapsulation efficiency was calculated as follows:

Encapsulation efficiency = (total nicotine content in formulation—free nicotine) × 100/total nicotine content in formulation

#### 2.2.5. Sustained Release Profile

The nicotine sustained release profile was analyzed using a vertical diffusion Franz cell, containing receptor and donator compartments and a cellulose membrane with 0.05 µm pores as a barrier. Phosphate buffered saline (PBS) was used as the receptor solution. This mixture was maintained at 37 °C during the entire time of the experiment. A volume of 500 µL of Eudragit nanocapsules containing nicotine was placed on top of the Franz cell, and, at the time intervals of 0.5 h, 1 h, 2 h, 4 h, 6 h, 12 h, 24 h, 48 h and 72 h after the beginning of the experiment, 1 mL of receptor solution was collected and 1 mL of fresh receptor solution was replaced in the receptor compartment. The collected samples were analyzed in regard to the nicotine concentration by the HPLC method.

#### 2.2.6. Nanocapsule Stability

A variety of nanocapsule suspensions were stored at 4 °C and protected from light. Immediately after preparation and after 7, 15, 30, and 90 days, the formulations were evaluated in terms of pH, particle diameter, polydispersity index (PdI) and zeta potential. Another set of suspensions were maintained at room temperature (25 °C) to evaluate if visible properties such as color and phase homogeneity would change in any manner.

### 2.3. In Vitro Treatment of Cells with Eudragit Nanocapsules Containing Nicotine

#### 2.3.1. Cytotoxic Effect and Cellular Viability

To assess the biosafety of the formulations, the nanocapsules used were subjected to a series of tests to evaluate their cytotoxicity and cellular viability. All the tests were performed in triplicate unless otherwise stated.

##### Cell Culture

PC12 cells (ATCC CRL-1721), an established neural cell model derived from a pheochromocytoma of the rat adrenal medulla, were cultivated in culture flasks with DMEM high medium supplemented with 10% fetal bovine serum inactivated by heat, 5% horse serum and 1% penicillin/streptomycin, and maintained in an incubator with a humidified atmosphere with 5% CO_2_ at 37 °C.

##### Nanocapsule Sterilization

Prior to every in vitro test, the nanocapsule formulations were produced following the above method and sterilized by UV light for 30 min.

##### Experimental Groups

In this experiment, for treatment with nicotine containing nanocapsules, two different concentrations of nicotine were used: 100 μM and 500 μM. Treatment of the control groups with empty nanocapsules was calculated based on the amount of Eudragit contained in the above concentrations.

The cell experiment was divided into seven groups:

1. Cells cultivated on a tissue culture plate without nanocapsule treatment (control);
2. Cells treated with 166 μg/mL of empty Eudragit nanocapsules (equivalent to group 4 concentration of Eudragit) (EN100);
3. Cells treated with 833 μg/mL empty Eudragit nanocapsules (equivalent to group 5 concentration of Eudragit) (EN500);
4. Cells treated with Eudragit nanocapsules containing 100 μM of nicotine (NNC100);
5. Cells treated with Eudragit nanocapsules containing 500 μM of nicotine (NNC500);
6. Cells treated with 100 μM of bulk nicotine dissolved in deionized water (NIC100);
7. Cells treated with 500 μM of bulk nicotine dissolved in deionized water (NIC500);

##### MTT Assay

Cell viability was evaluated by the Tetrazolium salt method (MTT), a colorimetric assay which uses the tetrazolium dye MTT 3-(4,5-dimethylthiazol-2-yl)-2,5-diphenyltetrazolium, which is reduced by the viable cells to its insoluble salt formazan, and which is purple in color. One thousand cells were plated and cultivated in 48-well plates, treated with nanocapsules and incubated for different time periods. After 1 and 7 days, the supernatants were removed and MTT reagent was added to the cell culture in the final concentration of 0.5 mg/mL. After 2 h of incubation at 37 °C, the MTT was removed and the formazan salts were dissolved in Dimethyl Sulfoxide (DMSO) and homogenized. Following this, the absorbance was measured by spectrophotometer at the wavelengths of 570 and 630 nm. The results were calculated by the difference of the values at the two wavelengths. Only viable cells are capable of reducing MTT, thus, elevating group absorbance after NC treatment.

##### 2LIVE/DEAD Assay

Cell viability was also evaluated by fluorescent staining with LIVE/DEAD kit (Life Technologies, Carlsbad, CA, USA), in accordance with the manufacturer’s protocol. This assay was performed 7 days after NC treatment.

#### 2.3.2. Cytokine Dosage

The IL-6, TNF-α and IL-10 cytokines were analyzed by the Enzyme-Linked Immunosorbent Assay (ELISA), following manufacturer recommendations (Boster Biological Technology, Pleasanton, CA, USA) at 1 and 3 days after treatment with the nanocapsules. Prior to the nanocapsule treatment, the PC12 cells were stimulated with 10 mg/mL of LPS for 18 h (lipopolysaccharide, Sigma Aldrich^®^, São Paulo, Brazil) in order to induce inflammation and then evaluate the NC anti-inflammatory effect.

A control group composed of cells cultivated in the tissue culture plate without LPS and nanocapsule treatments was added to these experiments.

#### 2.3.3. Statistical Analysis

Appropriate statistical tests were employed to evaluate the significance level achieved, in accordance with the respective methodology, using the software GraphPad 7.0. Values are expressed as means and standard deviation. Means comparisons were performed by analysis of variance (ANOVA) followed by the Dunnett post-hoc test. The significance value adopted was 5% (*p* < 0.05).

## 3. Results

### 3.1. Nanocapsule Characterization

#### 3.1.1. Macro and Microscopic Aspect

**Figure 1:**
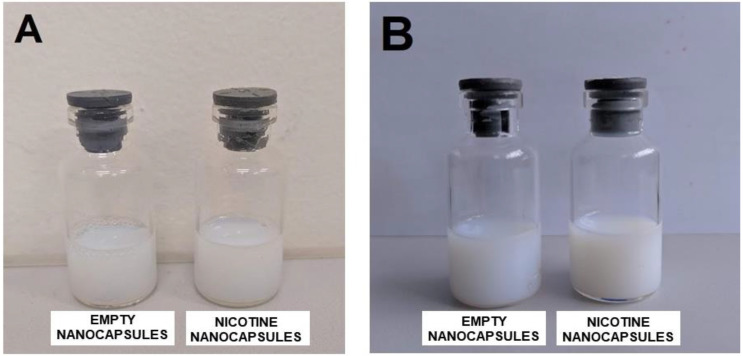
(**A**) Samples of freshly made Eudragit empty nanocapsules (left flask) and Eudragit nanocapsules containing nicotine (right flask); (**B**) samples of Eudragit empty nanocapsules (left flask) and Eudragit nanocapsules containing nicotine (right flask), 75 days after preparation, left at room temperature (25 °C).

Nanocapsule suspensions of Eudragit only or containing nicotine were prepared and appeared as white opaque fluids, similar to milk, with a blue reflex due to the Tyndall effect (Figure 1A depicts the aspect of freshly made nanocapsules, whereas Figure 1B shows the nanocapsules after 75 days of storage at room temperature, 25 °C).

**Figure 2:**
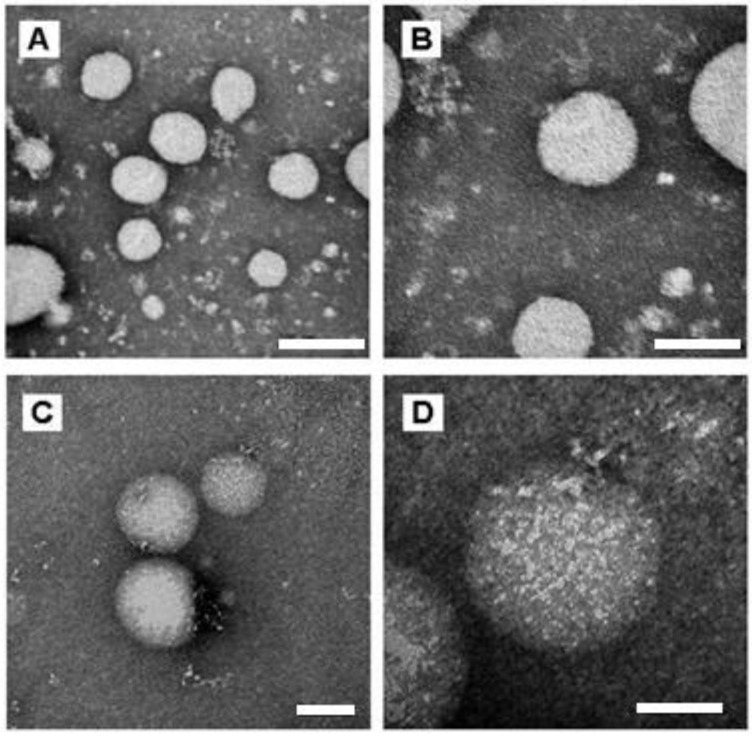
TEM images of empty Eudragit NCs (**A**,**B**) and Eudragit NCs containing nicotine (**C**,**D**) at different magnifications—(**A**,**C)** 150,000×; (**B**,**D**) 300,000×. Scale bars of (**A**–**C**) 200 nm (**D**) 100 nm.

To investigate the morphology of the NCs, transmission electron microscope (TEM) analysis was performed. As shown in Figure 2, both the empty nanocapsules and the nanocapsules containing nicotine presented spherical morphology, and their diameters were around 120 nm and 130 nm, respectively (Figure 2), which was further confirmed by ZetaSizer analyses (Table 1).

#### 3.1.2. Determination of pH

Freshly made Eudragit nanocapsules presented different ranges of pH, as demonstrated in Table 2.

#### 3.1.3. Encapsulation Efficiency

Encapsulation efficiency was calculated, as described in the materials and methods section. The value obtained was of 8.17% ± 0.47 (mean and standard deviation).

#### 3.1.4. Nanocapsule Stability

The nanocapsule suspensions were analyzed at five different time points: immediately after production—day 0, day 7, day 15, day 30 and day 90. The measured parameters were zeta potential (ZP), size (diameter), polydispersity index (PdI) and pH.

**Figure 3:**
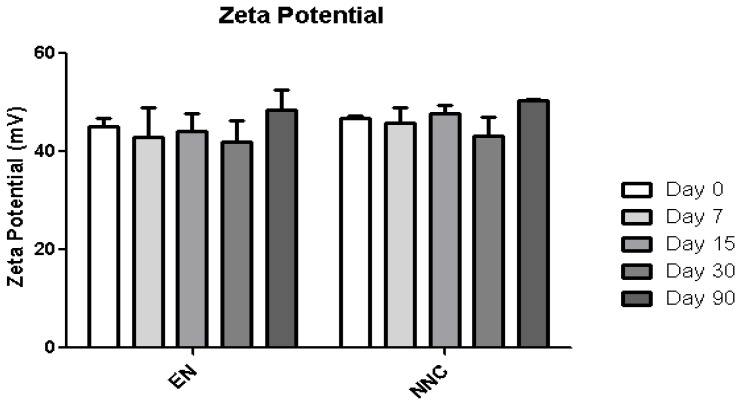
Zeta potential stability of empty nanocapsules (EN) or nicotine containing nanocapsules (NNC) for a period of 90 days of storage. No statistical difference was obtained, signifying that the zeta potential remained stable during the entire period of analysis.

**Figure 4:**
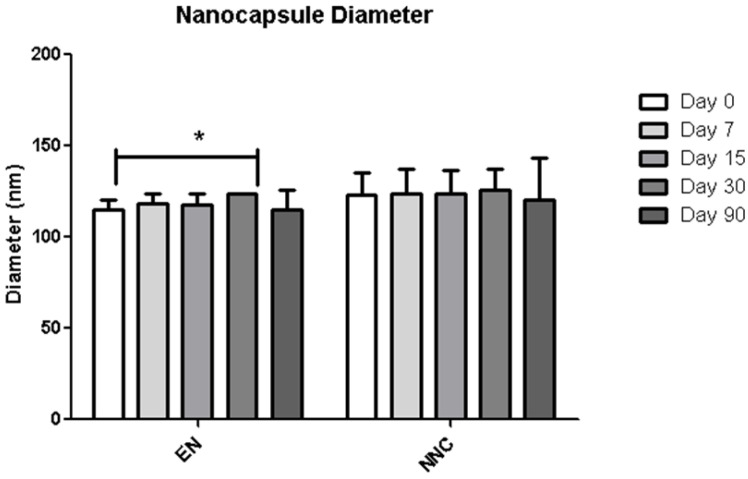
Distribution of diameter of empty nanocapsules (EN) or nicotine containing nanocapsules (NNC) for a period of 90 days of storage. Just a slight difference in size was observed between day 0 and day 90 in the empty nanocapsules, while the others remained stable throughout the period of analysis. *p* < 0.05-day 0 vs. day 60 (*n* = 3). *: *p* < 0.05.

**Figure 5:**
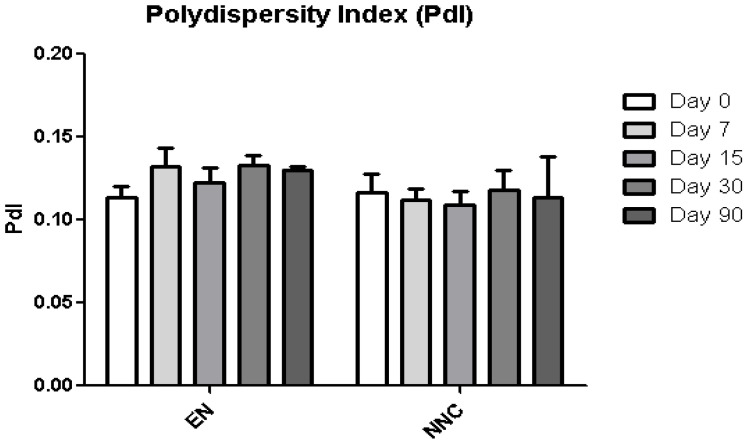
Polydispersity index (PdI) stability of empty nanocapsules (EN) or nicotine containing nanocapsules (NNC) for a period of 90 days of storage. No statistical difference was obtained, signifying that the size distribution of the nanocapsules remained stable throughout the period of analysis (*n* = 3).

Table 1 shows that both groups—nanocapsules with and without nicotine presented stable zeta potential values (Figure 3). The diameter also showed no change during the period of analysis (Figure 4), indicating that the nanocapsules did not aggregate or swell up, and their size distribution remained constant (Figure 5).

#### 3.1.5. Sustained Release Profile

**Figure 6:**
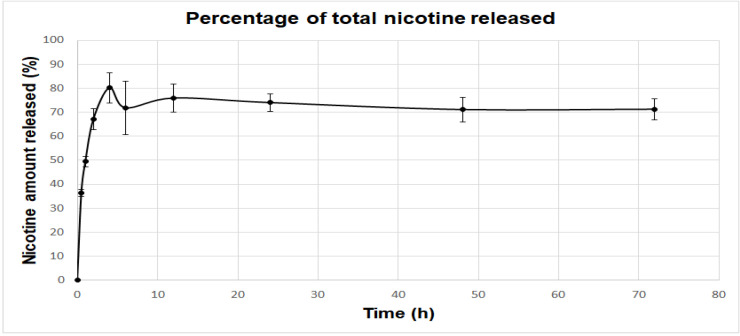
Percentage of the total amount of nicotine injected in the Franz Cell released at different time points. The initial release stage can be characterized as the “initial burst”, where the amount of nicotine that is adsorbed by the nanocapsule surface is released. The release profile then reaches a plateau of nicotine concentration. Results are displayed as mean and standard errors.

Eudragit nanocapsules containing nicotine presented a sustained release profile, as illustrated in Figure 6. This graph shows the percentage of the total amount of nicotine that was injected in the Franz Cell, which was released during the period of the experiment. There is the “initial burst”, the first release stage, where the amount of nicotine adsorbed by the capsule surface is primarily released. Following this, the concentration reaches a plateau.

### 3.2. Cell Viability

**Figure 7:**
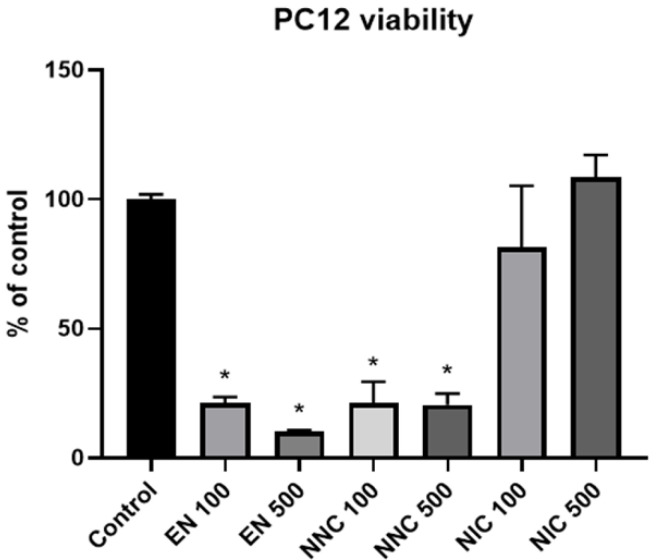
Viability of PC12 cells one day after various treatments with nanocapsules. EN 100 = empty nanocapsules (group 2); EN 500 = empty nanocapsules (group 3); NNC 100 = nicotine containing nanocapsules 100 μM (group 4); NNC 500 = nicotine containing nanocapsules 500 μM (group 5); NIC 100 = 100 μM of bulk nicotine (group 6); NIC 500 = 500 μM of bulk nicotine (group 7); (*: *p* < 0.05 samples vs. control), *n* = 3.

**Figure 8:**
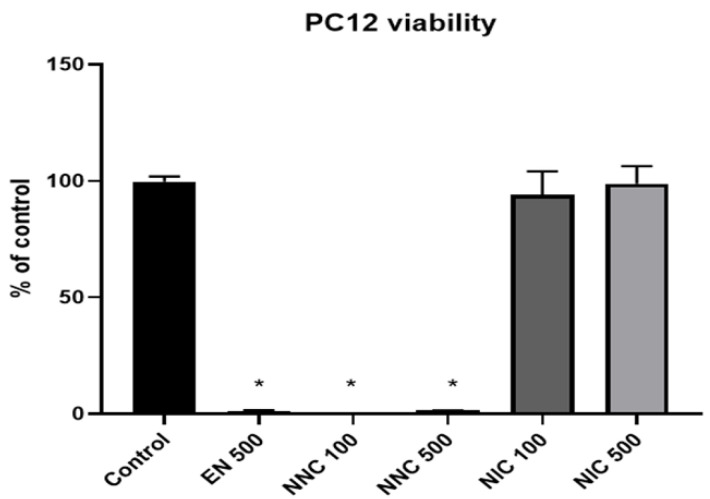
Viability of PC12 cells at seven days in culture. EN 100 = empty nanocapsules (group 2); EN 500 = empty nanocapsules (group 3); NNC 100 = nicotine containing nanocapsules 100 μM (group 4); NNC 500 = nicotine containing nanocapsules 500 μM (group 5); NIC 100 = 100 μM of bulk nicotine (group 6); NIC 500 = 500 μM of bulk nicotine (group 7); (*: *p* < 0.05 samples vs. control); *n* = 3.

PC12 cells treated either with empty nanocapsules or nicotine containing nanocapsules exhibited significantly lower viability compared to the cells that received no treatment (control group), or those which received treatment with bulk nicotine, as shown in Figure 7 (one day treatment) and Figure 8 (seven days treatment).

**Figure 9:**
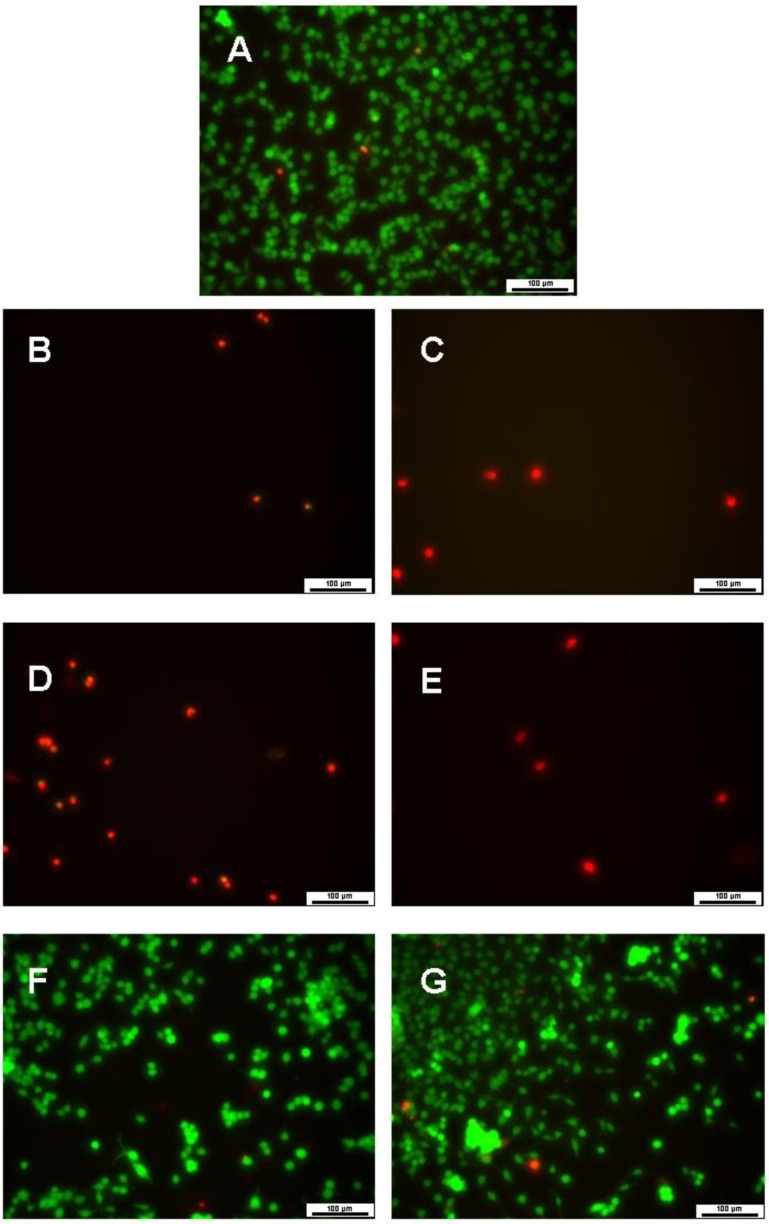
LIVE/DEAD images of PC12 cells 7 days in culture after treatment with nanocapsules. (**A**) control group; (**B**) EN 100; (**C**) EN 500; (**D**) NNC 100; (**E**) NNC 500; (**F**) NIC 100; (**G**) NIC 500 (*n* = 3 for all groups). Live cells are stained in green and dead cells are stained in red. Scale bars of 100 µm.

Cell viability at day 7 was also assessed by LIVE/DEAD assay (Figure 9), which corroborated the MTT results.

### 3.3. Cytokine Expression Evaluation

**Figure 10:**
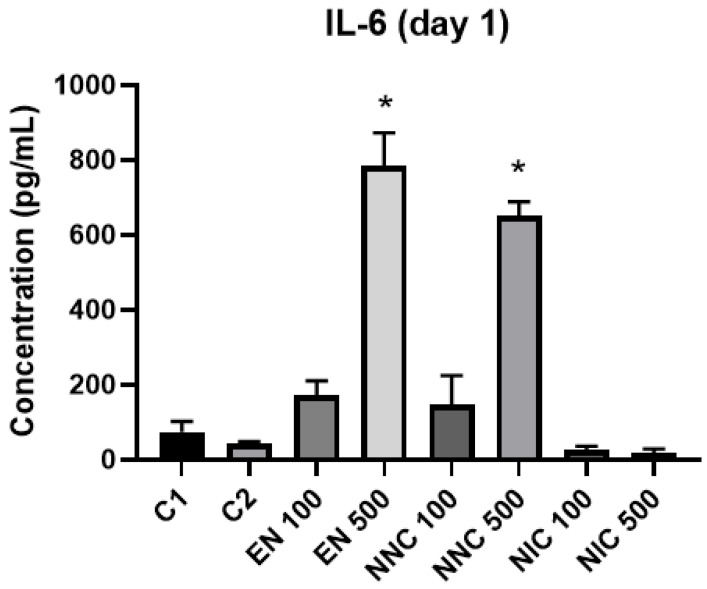
IL-6 detection one day after treatment with nanocapsules. C1 = control cells with no nanocapsule treatment and no LPS stimulus; C2 = control cells stimulated with LPS but no nanocapsule treatment. EN500 and NNC500 presented significantly higher levels of IL-6 compared to C1 (*: *p* < 0.05 samples vs. control).

**Figure 11:**
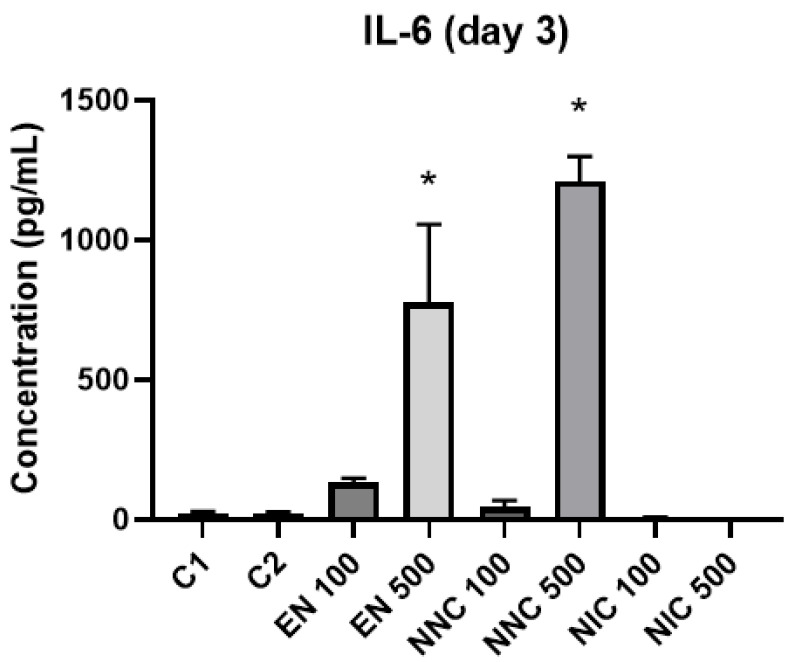
IL-6 detection three days after treatment with nanocapsules. C1 = control cells with no nanocapsule treatment and no LPS stimulus; C2 = control cells stimulated with LPS but no nanocapsule treatment. EN500 and NNC500 presented significantly higher levels of IL-6 compared to C1 (*: *p* < 0.05 samples vs. control).

The expression of cytokines TNF-α, IL-6 and IL-10 were evaluated by ELISA. IL-10 was not detected in our samples. At day one, IL-6 was significantly increased in the groups with higher empty nanocapsule concentration (EN500) and higher nicotine containing nanocapsule concentration (NNC500) (Figure 10). This increase is even more evident three days after treatment (Figure 11). Both groups of cells treated with bulk nicotine showed no difference in expression compared to the control groups.

**Figure 12:**
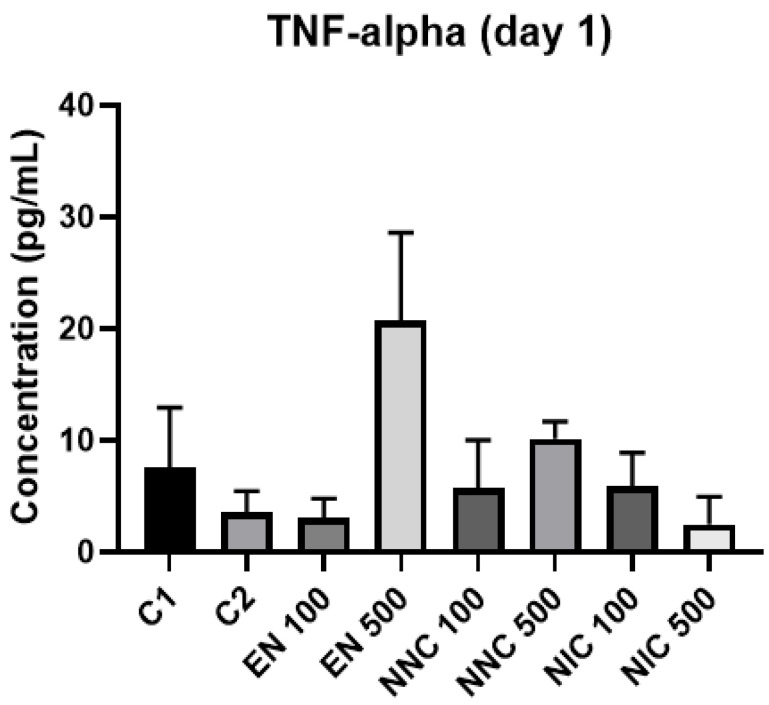
TNF-α detection one day after treatment with nanocapsules. C1 = control cells with no nanocapsule treatment and no LPS stimulus; C2 = control cells stimulated with LPS but no nanocapsule treatment.

**Figure 13:**
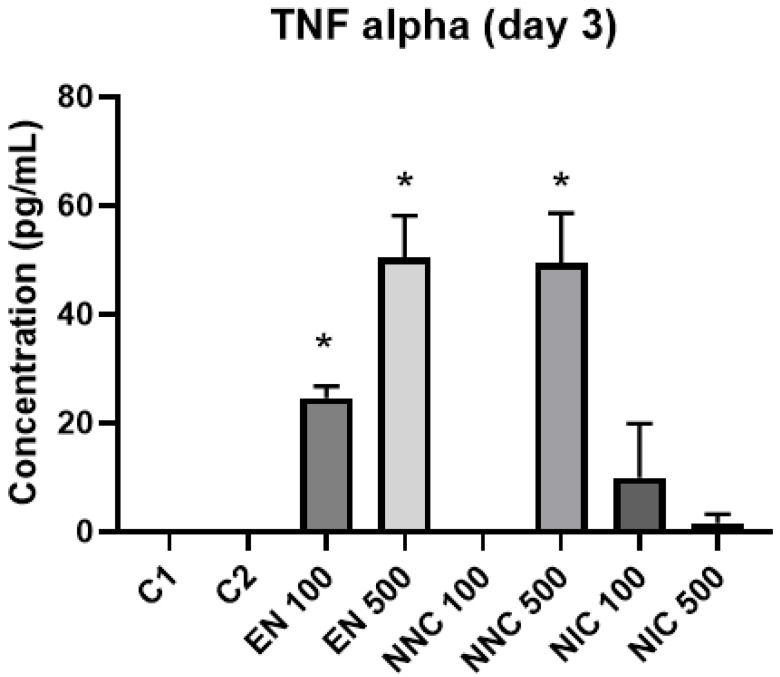
TNF-α detection three days after treatment with nanocapsules. C1 = control cells with no nanocapsule treatment and no LPS stimulus; C2 = control cells stimulated with LPS but no nanocapsule treatment (* *p* < 0.05, EN100, EN500, NNC500 vs. control).

At day one, TNF-α showed no significant difference in expression between the groups (Figure 12). However, at day three, all the groups treated with nanocapsules showed a significant increase of TNF-α expression, except for the group treated with Eudragit nanocapsules containing 100 μM of nicotine (NNC 100) (Figure 13). Both groups of cells treated with bulk nicotine showed no difference in expression compared to the control groups.

## 4. Discussion

In this study, nanocapsules of Eudragit containing nicotine were successfully produced and further characterized. According to the literature, the parameters for particle diameter obtained were satisfactory, around 120 nm for empty nanocapsules and 130 nm for nicotine containing nanocapsules, and for nanocapsule size distribution, values close to 0.1, meaning that most of the capsules had similar sizes in the gaussian curve. Zeta potential also had optimal values (around +45 mV for both formulations). The results show that the addition of the drug did not significantly change the structure of the Eudragit nanocapsules. The NCs also presented a good stability of the formulation when stored for 90 days, without any parametric and macroscopic alterations. These nanocapsule properties are in accordance with studies from Dalcin (2019) and Katzer (2014) and colleagues, who prepared Eudragit nanocapsules with the same interfacial deposition of the pre-formed polymer method [17,18]. The NCs containing nicotine showed a good, sustained release profile too, with an initial burst, that is to say, the release of the nicotine present in the surface of the capsule within the first 4 h of the experiment.

However, for this formulation, a poor encapsulation efficiency for nicotine was achieved, which means a low amount of nicotine (8.17% of the total added) was entrapped in the core of the nanocapsules. The encapsulation efficiency is highly dependent on the composition of the capsule core, such as the oil used, the characteristics of the drug encapsulated and the presence of surfactants that may facilitate the solubilization of the drug [3,19]. The fact that surfactants were not added to the organic phase in the nanocapsule preparation process might explain the low encapsulation efficiency obtained. Blouza and colleagues [20] suggest that the addition of a surfactant to the organic phase could increase encapsulation efficiency, but it could lead to a rise in nanocapsule size too, which is not desirable. It is therefore important to balance the amount of surfactant added to the formulation in order to reach a good encapsulation efficiency value, but also an appropriate particle diameter. Besides this, nicotine ditartrate dihydrate was used for the preparation of the Eudragit capsules, being a more hydrophilic salt, which may not dissolve well in an oily core. Using a more hydrophobic form of nicotine may enhance entrapment efficiency.

After their entrance in the living organism, nanoparticles are usually targeted by opsonization and suffer phagocytosis by monocytes and macrophages, ending up in different organs such as the liver, spleen and lymph nodes [21]. It is therefore important that, besides careful planning of the dose of drug loaded nanoparticles, it is also important to take in consideration the type of interaction between the cells and particles. Once inside the cells, nanoparticles can exert physical and chemical negative impacts, such as membrane damage, lipid peroxidation, production of reactive oxygen species (ROS), mitochondria damage, DNA damage, and protein misfolding, among others [22]. In this same manner, nanodevices developed for drug delivery can themselves induce pathological processes. Besides the cytotoxic effects, in the case of SCI, administration of nanocapsules directly into the already damaged spinal cord can generate more damage, such as occlusion, compression, inflammation and risk of infection. However, only a damaged spinal cord, that is, with a ruptured BBB, is capable of internalizing drug loaded nanoparticles [23].

In this study, it was demonstrated that Eudragit can be toxic to PC12 cells, as it decreased their viability and stimulated the production of pro-inflammatory cytokines. PC12 cells are a good model for toxicology testing, especially when dealing with nanoscience applications. The aim of the capsules produced in this study is for treating neuropathological disease. The decreased viability was unexpected; however, nicotine concentration was probably not the issue, as the cells treated with bulk nicotine presented viability rates similar to the control group. Moreover, nicotine poly(lactic-co-glycolic) acid nanoparticles containing similar concentrations of nicotine (100 µM) proved to be neuroprotective in a dopaminergic neurons culture [24]. It was shown by Larner and colleagues that various types of nanoparticles can be cytotoxic to this cell lineage [25]. In this study, concentrations of various types of nanoparticles above 100 µg/mL, similar to the concentrations used in our study, proved to be cytotoxic and increased vacuolization and membrane contraction [25]. The higher the concentration, the more cytotoxic it was to the cells that model brain tissue. Besides being a non-biodegradable polymer, Eudragit is commonly used for gastrointestinal administrations because of its mucoadhesive properties [26]. Dalcin and colleagues [17] had similar results when testing the cytotoxicity of the Eudragit nanocapsules on peripheral blood mononuclear cells, with a decrease in viability in a dose-dependent manner. Eidi and colleagues (2010) also showed that not only unloaded Eudragit nanoparticles, but also Heparin-loaded ones had a dose-dependent toxic effect in a macrophage cell line, being able to induce cell death as well [27]. These studies corroborate in the explanation of the cytotoxic effect of Eudragit on the PC12 cell line.

To evaluate the anti-inflammatory effect of nanocapsules containing nicotine, the expression of three different cytokines were quantified by ELISA after treating the PC12 cells: IL-6, TNF-α and IL-10. Cytokines are essential for the inflammatory process as they induce the expression of additional cytokines, chemokines, nitric oxide and reactive oxygen species [28]. The cytokine expression is a tightly regulated process: for instance, TNF-α and IL-6 are expressed in the initial stage of the inflammatory process, the effector phase. This may be the reason why the secretion of the two cytokines was elevated when compared to the control group on the first and third day of the study. The present results show that the treatment with high concentrations of Eudragit NCs induced inflammation. This may be due to the formation of the so-called protein corona [29]. The protein corona is a layer of proteins that bind to the surface of the particles when they are introduced in biological media [29]. Deng and collaborators (2011) also argue that the proteins that form this corona can interact with the integrin receptors on the cell membrane and, therefore, activate the NF-kB pathway and release pro-inflammatory cytokines [30]. The group of cells treated with the lower concentration of NCs containing nicotine showed a reduction of the expression of TNF-α, suggesting that the drug had attenuated the induced inflammation. On the other hand, IL-10 was not detected in the experiments. IL-10 is an anti-inflammatory cytokine and is responsible for shutting off the immune response, and so it is expressed in the attenuation phase of inflammation. As this cytokine was not detected, this data might explain why the cells presented high levels of pro-inflammatory cytokines [28]. However, assessment of cytokine expression in a single cell type is limited, given the full complexity of the in vivo immune response. This therefore requires further tests with different cell lines and a careful interpretation.

To improve NC cytocompatibility, one possibility is to functionalize the polymer and, thus, the surface of the nanocapsule to improve cell-particle interaction. In addition, the fact that Eudragit is a non-biodegradable polymer could also influence this interaction. Fujii and colleagues (2001) argue that the exposure of epithelial cells to non-biodegradable particles induces a pro-inflammatory state and the production of cytokines [31]. One study, for instance, which tested PLGA nanoparticles, a biodegradable polymer, showed that cell treatment with these particles did not increase expression of pro-inflammatory cytokines and thus, did not promote an induction of an inflammatory response [32].

Additionally, it is not uncommon for nanoparticles to form a sediment on top of the adhered cells instead of remaining suspended in the culture media, thereby increasing, the density of particles at the cell surface. It is speculated that the nanoparticle weight on top of the cells can influence processes such as cellular uptake and cell death [33,34], which may have occurred in our experiments, explaining the low viability presented by the cells treated with NCs.

Nevertheless, it is difficult to draw in vivo conclusions by in vitro tests, as the in vivo environment is much more complex: there is a greater number of cells and the presence of extracellular matrix and of circulatory systems, for example. Thus, it is vital to test this formulation in animal models, as understanding its pharmacokinetics is critical for gaining a better insight into its effects. In living organisms, nanoparticle toxicity is dependent on factors like organ distribution of the particle and retention times, which are mainly determined by nanoparticle features [35]. A study from Pereira and colleagues (2019), for example, demonstrated that unloaded Eudragit nanocapsules showed no toxic effects in two month old Wistar rats, including no oxidative stress and no hepatic damage markers [36].

Thereby, in future studies, it is our intention to improve the Eudragit nanocapsule formulation in order to achieve a better nicotine encapsulation efficiency value, lyophilizate the samples to increase their stability even further and reduce contamination risk; correct its pH to physiological values and, after further in vitro tests, perform in vivo experiments.

## 5. Conclusions

In this study it was possible to produce Eudragit nanocapsules containing nicotine that were satisfactorily characterized regarding their zeta potential, diameter, polydispersity index, pH and sustained release profile. The nanocapsules also presented good stability over time when stored. However, a low encapsulation efficiency was obtained, and this has to be further analyzed and improved. In vitro treatment of PC12 cells with nanocapsules showed that Eudragit was toxic to cells as it decreased their viability. Moreover, Eudragit induced inflammation in these cells as it elevated the expression of pro-inflammatory cytokines. More studies are therefore needed to better understand the cytotoxicity exhibited by this polymer.

## Figures and Tables

**Table 1:** Results for nanocapsule stability for 0, 7, 15, 30 and 90 days. ZP = zeta potential; PdI = polydispersity index; EN = empty nanocapsules; NNC = nicotine containing nanocapsules.

	**Day 0**			
	**ZP (mV)**	**Diameter (nm)**	**PdI**	**pH**
**EN**	45.02 ± 2.95	115.05 ± 7.24	0.113 ± 0.017	5.67 ± 0.288
**NNC**	46.77 ± 2.17	139.05 ± 18.85	0.116 ± 0.018	4.33 ± 0.288
	**Day 7**			
	**ZP (mV)**	**Diameter (nm)**	**PdI**	**pH**
**EN**	42.91 ± 9.08	118.17 ± 8.04	0.131 ± 0.021	5.67 ± 0.288
**NNC**	45.83 ± 4.73	123.45 ± 20.4	0.111 ± 0.015	4.33 ± 0.288
	**Day 15**			
	**ZP (mV)**	**Diameter (nm)**	**PdI**	**pH**
**EN**	44.16 ± 6.10	117.29 ± 9.25	0.122 ± 0.017	5.75 ± 0.25
**NNC**	47.56 ± 3.66	123.78 ± 18.5	0.101 ± 0.016	4.33 ± 0.288
	**Day 30**			
	**ZP (mV)**	**Diameter (nm)**	**PdI**	**pH**
**EN**	41.82 ± 7.19	118.35 ± 7.80	0.132 ± 0.014	5.83 ± 0.288
**NNC**	43.1 ± 5.93	125.9 ± 16.7	0.117 ± 0.02	4.16 ± 0.288
	**Day 90**			
	**ZP (mV)**	**Diameter (nm)**	**PdI**	**pH**
**EN**	50.83 ± 5.59	116.78 ± 9.81	0.114 ± 0.029	6.0 ± 0.0
**NNC**	51.44 ± 3.03	123.76 ± 20.49	0.107 ± 0.026	4.0 ± 0.0

**Table 2:** pH of freshly made empty nanocapsules (EN) and nicotine containing nanocapsule (NNC) suspensions. Results are displayed as mean ± standard deviation.

	Mean pH
**EN**	5.67 ± 0.288
**NNC**	4.33 ± 0.288

## Data Availability

The data presented in this study are available on request from the corresponding author. The data are not publicly available due to ongoing project that may be subject to a patent.
